# Bioengineering microbial communities: Their potential to help, hinder and disgust

**DOI:** 10.1080/21655979.2016.1187346

**Published:** 2016-05-24

**Authors:** Diane Sivasubramaniam, Ashley E. Franks

**Affiliations:** a Department of Psychological Sciences, Swinburne University, Melbourne, Victoria, Australia; b Department of Physiology, Anatomy and Microbiology, La Trobe University, Melbourne, Victoria, Australia

**Keywords:** artificial, community function, dread, function, gene circuits, genetic engineering, naturalness, synthetic biology, trust

## Abstract

The bioengineering of individual microbial organisms or microbial communities has great potential in agriculture, bioremediation and industry. Understanding community level drivers can improve community level functions to enhance desired outcomes in complex environments, whereas individual microbes can be reduced to a programmable biological unit for specific output goals. While understanding the bioengineering potential of both approaches leads to a wide range of potential uses, public acceptance of such technology may be the greatest hindrance to its application. Public perceptions and expectations of “naturalness,” as well as notions of disgust and dread, may delay the development of such technologies to their full benefit. We discuss these bioengineering approaches and draw on the psychological literature to suggest strategies that scientists can use to allay public concerns over the implementation of this technology.

## Introduction

Microorganisms are present in almost every habitat on earth. Microbes have shaped our planet over billions of years and are the drivers of global and local carbon, oxygen, nitrogen, sulfur, and phosphorus cycles that form the basis of life on our planet.^1^ The evolutionary processes that allow growth and survival of microorganisms across these varied environments have led to huge diversity among microorganisms and an enormous metabolic and genetic potential. No microorganisms exist in isolation, but all are part of complex communities comprised of many different individuals with different functions that form an active, dynamically changing community. While individual microbial species have been isolated from environments and studied *in vivo*, often the function of the microbe in the environment is more than the sum of individuals who habitat the area. Cells within a mixed community interact, communicate, influence and alter the biochemical and physiological processes among the members. This vast potential among microorganisms provides a tremendous environmental, industrial and agricultural potential to be used as a tool.

The simplest of natural communities still contains a wide range of species,^2^ with a vast majority of species still uncultured and uncharacterized. There is much interest in engineering these communities to allow a greater understanding of ecological and environmental processes, remove or reduce pollution and produce commodity products. The engineering of these communities has had many successes but still faces several challenges both scientifically and socially before their full potential can be realized.

### Understanding complex communities utilizing synthetically derived models

Microbes within mixed communities can undergo a wide range of interactions that can be broadly characterized into commensalism, competition, predation, cooperation, amensalism or no interaction at all.^3^ These interactions form complex webs that create difficulties in providing a mechanistic understanding of processes that occur. To overcome these difficulties, communities can be engineered to maintain key features of the natural communities that allow assessment of key ecological, evolutionary, structural and functional features under laboratory conditions. A well known synthetically derived model community was first created by Sergi Winogradsky using soil in a glass column in the 1880s.^4^ This community, enriched from the sediments and commonly called a Winogradsky Column, led to the discovery of chemolithotrophy due to stratification of the microbial community into unique environmental niches where the microbes were able to grow and be easily visualized due to pigmentation. These structured communities are capable of self-sustaining with only the input of light; all nutrient cycles such as carbon, nitrogen and sulfur cycles are carried out by the microbes within the column. Similar columns have since been used to study bioremediation, phosphate generation, bi-hydrogen and microbial community dynamics.^4,5,6,7^ This allows for the study of the communities in a top down approach investigating the complex interactions and function of a community as a whole. While removed from their natural habitat, the aim is to maintain the microbial community in a habitat as close as possible to their original. The complexity of ecosystems' dynamics can result in unintended results due to community function. Using acetate to stimulate dissimilatory iron reducing microbes in the subsurface has seen success in precipitating U(VI) by reduction to U(IV).^8^ When the acetate feed was ended, the reduced U(IV) could be used as an electron donor by the microbial community and was resolubilized. Understanding the redox reactions involved in this cycling has allowed the targeted solubilization/immobilization of Uranium in the environment through manipulation of the electron flow within the microbial communities.

Synthetic communities, microbial communities created from defined constituents, are greatly simplified to allow a bottom up approach for isolating specific interactions and providing in depth information from communities comprising as few as just 2 species. Numerous studies have followed the initial approach to reduce complexity to focus on specific interactions. The study of syntrophic interactions has benefited greatly by establishment of co-culture of the syntrophic partners to allow investigation of their interactions. Initial culturing was only possible with the use of both syntrophic partners in a 2-species community.^9^ Establishment of co-cultures undergoing syntrophic pressures to investigate the evolution of these interactions led to the discovery of direct interspecies electron transfer, a process whereby electron transfer does not require small molecules.^10^ These laboratory based synthetic communities led to direct interspecies electron transfer being studied within naturally forming complex communities of upflow anaerobic sludge blanket reactors and strategies, such as use of activated carbon as an electron conduit, to enhance the activities of these communities during treatment of industrial organic wastewaters.^11,12^ Through the simplification of the bioengineered community, mechanistic studies examining interactions on the genetic, protein and metabolic levels provide insights that could not be initially done in a complex community.

### Bioengineering microbial communities using interactions

Microbial communities undergo a number of interactions, the most significant being metabolic, that need to be understood if desired outcomes are to be achieved. These interactions are outlined in Table 1 and cover a range of processes occurring in the community. Interdependencies, both positive and negative, are important considerations in defining communities as they define multidimensional interactions and promote species richness as well as bolsters community stability.^13^ These interactions often involve evolutionary pressures leading to changes in fitness and causing long-term prediction and modeling difficulties.

**Table 1.** Basic motifs of microbial interactions as outlined in [3]. Effect is designated as either (0) no effect, (−) negative effect or (+) positive effect for the microbial partners in the community.

Effect	Ecological interaction	Metabolic
0/+	Commensalism	Food Chain
−/−	Competition	Substrate Competition
−/+	Predation	Food chain with waste
0/0	No interaction	No common metabolites
+/+	Cooperation	Syntrophy
0/−	Amensalism	Waste product inhibition

One of the most important bioengineered communities currently in widespread use is wastewater treatment. Wastewater treatment plants exist worldwide with mixed inputs, but all with the aim of treating wastewater and removing contaminants. Often, open systems involving microbial populations can be difficult to predict and seem to occur almost at random in their initial stages.^14,15^ Since wastewater treatment has a functional goal (i.e., the removal of organic compounds, nitrogen, phosphorus and toxic products), the process is not defined by a specific microbial species but functional units. These functional units are of great interest for improvement of waste treatment, especially since a large majority of the microbial species remain unculturable. Since the biological activity determines the rate and efficiency of treatment, recognizing structure and functional groups indicative of productivity is of great benefit to maintaining an efficiently operating wastewater treatment plant. While not a new idea, anaerobic digestion has become of increasing interest to recoup energy in wastewater streams by converting biological waste into biogas (a mixture of methane and carbon dioxide). Previous mechanical limitations have been investigated and improvements had been sought; reactor design, mixing efficiency, buffering and multiple step digestion processes have been seen as engineering solutions to improve efficiency.^16^ These factors are now being investigated in terms of microbial community and growth with the use of industrial conditions that produce efficient cost-input/output ratios. The establishment of model systems that improve the link between laboratory scale experiments and transition into industrial scale setup in terms of improving stability and efficiency, as well as providing predictive community level function, are recommended as a future way of advancing waste water treatment through bioengineering naturally occurring communities.^17^

Electromicrobiology is now providing the ability to add energy into a community in a specifically targeted manner.^18^ The use of poised electrodes as either electron donors or acceptors has allowed the stimulation of specific biological pathways in the community due to an electrode acting as an electron acceptor or donor at a specific redox potential that also enables the exclusions of others. Furthermore, only organisms that can interact with an electrode are stimulated, allowing a focus on specific subset of the microbial community without stimulation of a broad cross section that may occur with organic amendments.^19^ A wide range of bioremediation and bioproduction capabilities have been demonstrated with microbial electric systems and are expected to become of more interest in the future.^20^ Interestingly, stability can be increase in anaerobic digesters when electrodes are included by biomass retention and maintaining community diversity, and may even act as a microbial tool to correct failing anaerobic digesters.^21^ A further advantage of electricmicrobes is the ability to combine synthetic biology principals to engineer strains for the bioproduction of commodity chemicals using CO_2_ as a carbon source.

### Synthetic biology bioengineering microbes for industrial production

Biorefinery is seen as a key concept in the development of industrial biotechnology.^22^ The biorefinery is proposed to produce a number of different commercial products, completely utilizing the input substrate(s) and producing a variety of commercial products, analogous to a current petroleum refinery. Traditional bioproduction utilizing pure cultures has focused on a single product, often with large success when the product is of large commercial value. Insulin production in *E. coli* is an example of an early success in engineered pure cultures.^23^ Microbial production, even in engineered strains, often cannot be achieved using a single strain, being limited by metabolic load, need for optimization and the total manipulation that can be made to a single cell or pure culture.

Synthetic biology has the goal of overcoming these limitations by reducing the microorganism to a programmable chassis whose functions can be predicted using standardized DNA building blocks.^24^ To reach these stated goals a number of important issues have to be addressed. Many engineered gene circuits have biological uncertainties that cause loss of predictive ability when applied to differing microbial hosts.^25^ To overcome the need for using separate microbial hosts, a custom built microbial factory using a minimum microbial genome is being developed. This will produce a microbial chassis that can be utilized to build a synthetic cell allow quantification of biological components in a predetermined manner.^26^ To achieve integrated circuit design, steps have been taken to what has essentially been described as a programing language for the design of computational circuits in living cells.^27^ These circuits contained on plasmids could provide specific cellular response to multiple environmental inputs or provide timing of gene expression in a desired fashion. However, construction of even simple circuits is still currently time-intensive and unreliable.^27^

Synthetic biology has the potential to provide access to a huge range of diverse complex molecules with a wide range of applications. Environmental and production constraints can be removed through design and new and novel compounds produced. Synthetic biology has the promise to reinvigorate drug discovery pipelines and is stimulating a range of bioengineering tools.^28^ Synthetic biology is providing a greater understanding of natural product gene clusters and the ability to synthesize DNA at reduced costs,^29^ genetic refactoring to convert multigene systems into programmable “parts,”^30^ advancement on regulation and genetic optimization between hosts,^31^ as well as multiplexed genome editing with CRSIP-9.^32^ Microbial production of a range of compounds has already been initiated with some becoming commercially available such as anti-malarial FR900098, natural and semisynthetic opiods, pristinamycin II and bisindole, among others.^28^ The decrease in cost of DNA syntheses is opening up the possibility to truly program microorganisms to produce a range of products that can be utilized in medicine, industry and agriculture.

### Acceptability of biotechnology

While it is important to establish the biotechnology that is *possible* in microbial communities, it is also important to establish the conditions under which biotechnology is *acceptable* in human communities. Citizens' scepticism about agricultural biotechnology has driven consumer backlash, which has led to financial losses for agricultural biotechnology organizations and delays in regulatory approval of GM crops.^33^ The palatability of agricultural biotechnology to the public has serious consequences for the viability of the science: while some governments believe that at least some of these technologies pose no risk to people or the environment, they are prevented from developing these technologies by public hostility.^34^ In this section, we review the psychological research on human perceptions of biotechnology, using genetic modification of food crops as a model for understanding potential responses to bioengineering microbial communities.

### Sanctity of nature

Several studies have demonstrated that people's attitudes to GM foods are not determined by a simple calculation of potential risks and benefits, but are driven by several factors.^34^ One key factor shaping attitudes to biotechnology is a reverence for things that are perceived as “natural” – that which is “natural” is seen as good.^35^ Researchers have referred to this as a “natural preference,” encapsulating people's preference for natural entities over those that have been produced with human intervention, and this preference occurs particularly in the domain of foods.^36^ Furthermore, laypeople's definitions of “natural” are largely a rejection of scientific intervention: when people are asked to define the term “natural,” they do so principally by the absence of certain negative features (such as additives, pollution, and human interference) rather than by the presence of any particular positive features.^35^

Human concerns about interfering with a natural entity can be divided into 2 categories: instrumental and ideational. Instrumental concerns are essentially an assessment of risk and benefit, and are centered on the view that preservation of the natural world is ultimately better (less risky and/or more beneficial) for the success and even survival of the human race. There are specific advantages to natural entities – they are thought to be healthier, more appealing, or kinder to the environment than non-natural entities. Ideational concerns operate independent of concerns for human welfare: the ideational view posits that the original form is, by its nature, the best form, and it is morally correct to preserve and defend the natural world. The natural entity is preferred because it is simply “better” – more moral, more beautiful, or simply “right.”^35,36^ For instrumental or ideational reasons, therefore, people respond negatively to what they see as interference with a natural entity, and the particular emotional response that people display will depend on whether their concerns about the interference are instrumental or ideational.

Ideational concerns involve a moral element – the preservation of nature as a moral imperative – and psychologists have established that the emotional response elicited by moral violations is disgust (e.g. ^37^). In particular, disgust links with moral concerns that involve sanctity, divinity, and the protection of what are seen as sacred objects and values. To the extent that nature is seen by people as sacred and morally “right,” the violation of natural entities (through, for example, additives, pollution, or human interference) will prompt disgust responses.

The large body of research on risk decision making shows that people respond to instrumental concerns about potential hazards along 2 dimensions: dread and uncertainty. Dread includes perceptions of lack of control over a hazard and catastrophic potential fatal consequences. Uncertainty refers to hazards being unobservable, unknown, new, or delayed in their potential harm.^38^ While experts often define risk in terms of mortalities per year, laypersons often include other factors, closely linked with dread and uncertainty (e.g., catastrophic potential, voluntariness, effects on future generations, and controllability), in their determinations of risk. This discrepancy between lay and expert assessments of risk leads to lay people assigning little weight to expert assessments when determining their own degree of instrumental concern over biotechnology.^39^

A key finding in the psychological literature regarding responses to biotechnology is the discovery that people respond more strongly to the *process* by which entities are modified than they do to the content of those entities.^36^ The insertion of a gene from another species produces the largest drop in perceived “naturalness,” even though this process produces minimal change in the entity's genotype and phenotype. In contrast, domestication, a significant human intervention that changes the genotype and phenotype of an entity in major ways over hundreds of generations, is perceived by laypeople to be much less damaging to the “naturalness” of an entity. Based on these and other findings (e.g.,^40^), researchers have surmised that the primary basis for opposition to genetic modification is ideational, and have speculated that what appears critical is the level at which humans intervene: it may be the case that the notion of interfering with nature is highlighted more clearly by human insertion of a single gene than by the artificial selection that occurs in domestication.^36^

### Factors moderating human responses

There are certain factors that predictably affect people's perceptions and responses to biotechnology. For example, education and greater knowledge of science increase support for genetic engineering.^34,35^ Some religious groups emphasize that crossing species is unnatural^41^ and religiosity significantly impacts attitudes to genetic modification (though it does not impact attitudes to other environmental issues^42^).

A crucial factor affecting people's support for biotechnology is trust in the institution that is developing and implementing the technology. Trust helps people to reduce uncertainty to an acceptable level and simplify decisions involving a large amount of information; it is particularly important, therefore, when people's knowledge about a topic is low,^43,44^ or when claims about safety are fervently contested, as is the case with GM food.^45^ Unfortunately, there are low levels of trust in some of the scientific experts and institutions that are charged with developing and implementing science-based policy and practice, and this low confidence is widely attributed to highly publicized controversies in recent years, such as that surrounding bovine spongiform encephalopathy (BSE).^46^ There are differences in the level of public trust enjoyed by different institutions, with evaluators (e.g., scientists) the most trusted, watchdogs (e.g., environmental organizations) somewhat trusted, and industry and government least trusted.^47^

The type of biotechnology in question also shapes responses. As noted above, process is more important than content in shaping people's attitudes to genetic modification: even though it involves far more extensive changes to genotype and phenotype, people are more supportive of domestication than genetic engineering.^36^ When genetic modification does occur, people are more supportive of cisgenesis (the artificial combination of genetic elements derived from the same species) than transgenesis (the artificial combination of genetic elements derived from different organisms that cannot be crossed by natural means^34^). Taken together, the data suggest that genetic modification is tolerated to the extent that it could have successfully occurred without human intervention (i.e., to the extent that the genetic modification could have conceivably occurred in the natural environment).

### Scientists' response

To some extent, time itself will eradicate some community opposition as people's familiarity with bioengineered communities grows: researchers have noted that attitudes to “natural” are dynamic, and the definition of “naturalness” changes over time (for example, in the 19^th^ century, “natural product” primarily referred to a perishable product^35^).

Beyond waiting for the public's familiarity to grow, however, the psychological literature suggests some proactive measures that scientists and policy makers can undertake, to allay public concerns about biotechnology involving bioengineering. As noted above, opposition to genetic modification is both instrumental and ideational – it is therefore important to counter both of these avenues of opposition. While instrumental concerns can be countered by educating the public about the risks and benefits of biotechnology (with appropriate attention to people's dread and uncertainty responses as outlined above), scientists must also address the public's moral concerns about interfering with nature. Recently, food security has re-emerged as a global issue, as the specter of food shortages is raised by population growth, increasing demand from more affluent countries, and climate change.^33^ In this light, agricultural biotechnology that contributes to food security presents a moral imperative of its own, and focusing on this message allows scientists to address the ideational, as well as the instrumental concerns of the public (though important concerns have been raised about the manipulative use of this food security framing in the GM debate; for a review, see ^33^).

Finally, scientists and policy makers should be aware that one factor affecting the acceptability of risk is voluntariness: voluntarily chosen risks are perceived as more acceptable than those that are imposed.^48^ Therefore, to the extent that people voluntarily consume foods produced with various forms of agricultural biotechnology, we can expect that they will view the risks associated with those technologies as acceptable. Various mechanisms could be employed to ethically increase the voluntariness of public consumption of bioengineered food, such as public involvement in discussions about biotechnology policy and practice.^46^ Labeling is another important mechanism: some research has shown that labeling of an artificially modified product reduces the perception of risk associated with that product, regardless of whether consumers actually purchase that product.^49^

## Conclusions

The bioengineering of microbial communities for agricultural and industrial uses has great potential. Current research utilizing top down and bottom up approaches is providing insights and important mechanistic understanding of processes and interactions that may be exploited in the environment. Electromicrobiology is allowing the targeted input of energy to drive these processes *in situ*, exploiting naturally occurring microbial communities. In an almost opposite approach, synthetic biology is removing complexity to engineering microorganisms that are programmable for specific tasks. Advances are making these applications more useful with products being produced for market. Interestingly, there is still a divide between acceptance of these different approaches to bioengineering microbial communities for beneficial process and products. The psychological literature leads us to conclude that people will find cultivation of microbial communities more palatable when it is done via a process of natural selection *in situ* than through modification at the genetic level. If genetic modification is employed, the species from which genes are derived will be important in determining public support, as will the characteristics of the public audience (e.g., level of education, scientific knowledge, religiosity, and trust in the organization(s) managing the biotechnology). The psychological literature also suggests some proactive mechanisms by which we can make biotechnology more palatable to the human communities in which it will be applied. Understanding these human responses to biotechnology can inform our strategies for cooperation and communication with the public, better enabling us to allay any unnecessary fears held in the community.
